# Ultrasonic Aspiration-Acquired Glioblastoma Tissue Preserves Lymphocyte Phenotype and Viability, Supporting Its Use for Immunological Studies

**DOI:** 10.3390/cancers17040603

**Published:** 2025-02-11

**Authors:** Eftychia Stavrakaki, Zineb Belcaid, Rutger K. Balvers, Lisette B. Vogelezang, Wouter B. L. van den Bossche, Demi Alderliesten, Karishma Lila, Thierry P. P. van den Bosch, Jacques J. M. van Dongen, Reno Debets, Cristina Teodosio, Clemens M. F. Dirven, Martine L. M. Lamfers

**Affiliations:** 1 Department of Neurosurgery, Brain Tumor Center, Erasmus University Medical Center, 3015 GD Rotterdam, The Netherlands; e.stavrakaki@erasmusmc.nl (E.S.); z.belcaid@erasmusmc.nl (Z.B.); r.balvers@erasmusmc.nl (R.K.B.); l.b.vogelezang@umcutrecht.nl (L.B.V.); w.vandenbossche@erasmusmc.nl (W.B.L.v.d.B.); c.dirven@erasmusmc.nl (C.M.F.D.); 2 Department of Pathology, Erasmus University Medical Center, 3015 CN Rotterdam, The Netherlands; d.kortekaas.1@erasmusmc.nl (D.A.); t.vandenbosch@erasmusmc.nl (T.P.P.v.d.B.); 3 Cancer Research Center (IBMCC, Instituto de Biología Molecular y Celular del Cáncer, CSIC—University of Salamanca), Cytometry Service, NUCLEUS, 37007 Salamanca, Spain; j.j.m.vandongen@eslho.org (J.J.M.v.D.); cristina.teodosio@usal.es (C.T.); 4 Department of Immunology, Leiden University Medical Center, 2333 ZA Leiden, The Netherlands; 5 Laboratory of Tumor Immunology, Department of Medical Oncology, Erasmus MC Cancer Institute, 3015 GD Rotterdam, The Netherlands; j.debets@erasmusmc.nl

**Keywords:** glioblastoma, immunophenotyping, lymphocytes, resection, spectral flow cytometry, T cells, ultrasonic aspiration

## Abstract

**Background and Objective**: Access to high-quality patient-derived brain tumor tissues is instrumental for translational neuro-oncology research. Glioblastoma tumor material resected by ultrasonic aspiration (UA) during surgery offers an abundant source of material; however, it is generally not used for research experiments. We hypothesize that UA-derived tumor tissue represents a source of tissue that accurately reflects the immune infiltrates of glioblastomas. **Methods**: In this study, we have utilized UA-derived tissue and performed a head-to-head comparison with paired resection tissue from the vital tumor core of the same patient. A combination of 16 fluorochrome-conjugated antibodies was designed to identify tumor-infiltrating T, B, and NK lymphocytes and characterize the TILs by spectral flow cytometry. Furthermore, a 5-plex panel was designed to spatially characterize the T cells, macrophages, and tumor cells on the paired UA and resection tissues. **Results**: UA-obtained cells exhibited a comparable yield and viability, as well as an abundance of tumor-infiltrating T, B, and NK lymphocytes compared to resection sample-derived cells. Importantly, we observed that there is a high concordance with respect to expression intensities of immune checkpoints by T cells in both types of tissue samples. **Conclusions**: These findings underscore the feasibility and reliability of utilizing the immune infiltrates from ultrasonic aspiration-acquired glioblastoma tissue.

## Simple Summary

Glioblastoma is the most common and aggressive brain cancer, with a poor survival rate of about 15 months despite current treatments, which include surgery, radiation, and chemotherapy. During surgery, neurosurgeons often use a technique called ultrasonic aspiration (UA) to safely remove tumor tissue. This technique generates a large amount of material, much of which is typically discarded. This study explored whether UA-derived tissue could serve as a reliable resource for research, particularly for studying immune cells in glioblastomas. By comparing UA-derived tissue with resection samples, which are the standard tumor tissue samples, we found that UA tissue contained similar quantities and types of immune cells, such as T, B, and NK lymphocytes. These immune cells were viable, functional, and closely resembled those in resection samples. These findings demonstrate that UA-derived tissue is a valuable and abundant resource for advancing our understanding of glioblastoma’s immune microenvironment.

## 1. Introduction

Glioblastoma is the most common primary brain cancer, which has a poor prognosis of 15 months overall survival despite maximal adjuvant therapies. Standard of care consists of surgical resection or biopsy followed by radiation therapy and chemotherapy [1]. If tumor debulking of suspected glioblastoma is feasible, it not only enables cytoreduction but also offers the necessary tissue for diagnosis. Tumor debulking is meticulously performed with a primary focus on safety, aiming to minimize the residual tissue to the greatest extent possible, as this has been shown to improve prognosis [2]. To achieve this goal, ultrasonic aspiration (UA) is often used since it provides the opportunity to gradually advance the resection in a controlled manner into the invasive border of the tumor that intermixes with normal brain parenchyma [3]. As a result of this gradual circumferential reduction in tumor tissue, an abundance of tissue cells intermingled with rinse fluid and blood are disposed during surgery into the UA reservoir. The contents of this reservoir are often discarded or sometimes used for routine pathology or research objectives.

Despite advances in cancer research in the past decade, there have been no major improvements in outcome for patients with glioblastoma. While immunotherapies have demonstrated success in treating other types of solid cancers, their sustained therapeutic benefits remain elusive for the majority of glioblastoma patients [4]. This can be attributed, at least in part, to the unique microenvironment of glioblastoma, which impacts the efficacy of immunotherapies such as checkpoint inhibition [4,5]. As a result, there has been an increased focus on understanding the intricate interplay between the immune compartment within the microenvironment, particularly T cells, and tumor cells.

Hence, it is imperative for researchers to access patient tissues that can facilitate a comprehensive investigation of these aspects. UA material could emerge as a valuable alternative to the comparatively limited resection samples, potentially offering a rich source of material for investigation. However, it is crucial to assess whether UA-derived samples accurately represent the glioblastoma immune microenvironment and can substitute for resection samples. Previous studies have demonstrated the viability of UA-derived fragments for various applications, including cell culture [6], xenograft implants [7] and RNA sequencing [8]. While UA-derived lymphocytes are known to be viable and functional [9], it remains unexplored whether T cells from corresponding locations in resection and UA tissues exhibit the same characteristics and immunophenotype. We addressed this by comparing T cells using multi-parameter flow cytometry and multiplex immunohistology techniques in paired UA and resection tissues from the non-necrotic core of the same tumor.

## 2. Methods

### 2.1. Patients and Tumor Tissue Processing

Glioblastoma tissues were collected with the patients’ informed consent by the Department of Neurosurgery at Erasmus University Medical Center (Rotterdam, The Netherlands) (Supplementary Table S1). The collection of tumor tissue was approved by the institutional review board and in accordance with institutional and national regulations. To avoid differences due to spatial heterogeneity of the tumors, pairs of UA and resection tissue were obtained from contrast-enhancing areas of isocitrate dehydrogenase 1-wild type (IDH-WT) glioblastomas, which showed viable tumor tissue during surgery under microscopic vision. The ultrasonic surgical aspirator utilized by the neurosurgeons to collect the UA tissue specimens of this study is Integra CUSA Excel+ (Princeton, NJ, USA). The resection material was collected directly from the operation theater in DMEM (Gibco, Thermo Fisher Scientific, Bleiswijk, The Netherlands) with 1% penicillin/streptomycin. In contrast, the UA tissues were temporarily stored in the collection sock until transported to the laboratory (Supplementary Figure S1) and then placed in DMEM (Gibco, Thermo Fisher Scientific) with 1% penicillin/streptomycin. Subsequently, each tissue specimen was divided into two parts: one immediately snap-frozen at −80 °C, and the other collected for subsequent isolation of tumor-infiltrating lymphocytes (TILs). Both types of tissue specimen were processed according to Balvers et al. to obtain single-cell suspensions [10]. Briefly, the tissue from each tissue source was mechanically dissociated with a scalpel, and subsequently enzymatically dissociated with 2 mg/mL collagenase A (Sigma-Aldrich, Amsterdam, the Netherlands) and 0.15 mg/mL DNase I (Sigma-Aldrich) in DMEM supplemented with 1% penicillin/streptomycin at 37 °C for 1 h. The resulting cell suspensions from both tissue sources were filtered through a 70 µm cell strainer and were incubated in erythrocyte lysis buffer (155 mM NH4Cl, 0.1 mM EDTA and 10 mM KHCO_3_ in Milli-Q water) for 10 min at 4 °C for red blood cell removal. Subsequently, cells were washed twice in phosphate-buffered saline (PBS) and once with PBS containing 0.5% bovine serum albumin, 0.1% sodium azide, and 2 mM EDTA for further processing.

### 2.2. Spectral Flow Cytometry

Panels of 16 fluorochrome-conjugated antibodies (Supplementary Table S2) were designed to quantify and characterize the TILs derived from UA and resection tissues. Samples were processed and stained according to the standardized EuroFlow protocols (www.euroflow.org/protocols, accessed on 1 September 2021) [11]. All incubations were performed in the dark. Briefly, 10 × 10^6^ single cells deriving from resection and 10 × 10^6^ deriving from UA were stained with the antibody panel for 30 min at room temperature, washed with PBS, and incubated with a viability marker (LIVE/DEAD™ Fixable Blue Dead Cell Stain Kit, for UV excitation, Thermo Fisher Scientific) for 30 min at 4 °C. Subsequently, cells were fixed according to the manufacturer’s protocol (eBioscience™ Foxp3/Transcription Factor Staining Buffer Set). Finally, cells were washed with 1X Permeabilization Buffer three times and resuspended in 500 µL of PBS for data acquisition with the 5-Laser Aurora spectral flow cytometer (Cytek Biosciences, Wuxi, China). Data analysis and canonical multivariate analysis were performed with Infinicyt™ software 2.0.6 (Cytognos S.L., Salamanca, Spain). The quantification of viable T cells per gram of tissue was achieved by dividing the cell count per gram of the corresponding tissue by the percentage of CD3+ T cells identified within the same sample.

### 2.3. Automated Multiplex Immunofluorescent Staining

To spatially profile immune cell subsets in resection and ultrasonic aspirate (UA) tissues, we employed automated multiplex immunofluorescence (IF) staining using the Ventana Benchmark Discovery system (Ventana Medical Systems Inc., Roche, Tucson, AZ, USA). Our selected panel consists of five antibodies targeting T cells (CD3, PD1, HLA-DR), macrophages (CD68, HLA-DR), and tumor cells (Glial fibrillary acidic protein (GFAP), followed by DAPI counterstaining for nuclei (Supplementary Table S3). The multiplex IF protocol included five serial antibody staining steps, encompassing deparaffinization, heat-induced antigen retrieval, and antibody incubation, detection, and visualization steps for each marker. Following the staining sequence, sections were incubated in PBS with DAPI and covered with anti-fading medium. Slides were scanned with a ZEISS Axio Imager 2.0 fluorescence microscope, ZEISS, Breda, Netherlands (40× magnification), and the images were analyzed using QuPath software (version 0.4.3). The analysis focused on five manually selected regions of interest, utilizing the cell detection command for nuclei identification and applying a simple threshold method to classify each channel and calculate the number of positively labeled cells normalized per mm^2^ area [12].

### 2.4. Statistical Analysis

For statistical comparisons, the non-parametric paired Wilcoxon *t*-test was used to compare groups. Two-way ANOVA was used for immunofluorescence quantification data comparison. Statistical significance was considered at *p* < 0.05. Statistical analyses were performed using Graphpad Prism 9.

## 3. Results

### 3.1. UA-Derived Tissue Is an Abundant Source of Tumor-Infiltrating T, B, and NK Lymphocytes

In this study, we investigated whether UA-derived T cells are a suitable alternative for resected-derived T cells. Given that neurosurgeons commonly employ a combination of both techniques, we quantified the total material acquired through ultrasonic aspiration and resection. Our findings demonstrated that the total material obtained from ultrasonic aspiration generally surpassed that obtained from resection (Supplementary Figure S2).

**Figure 1:**
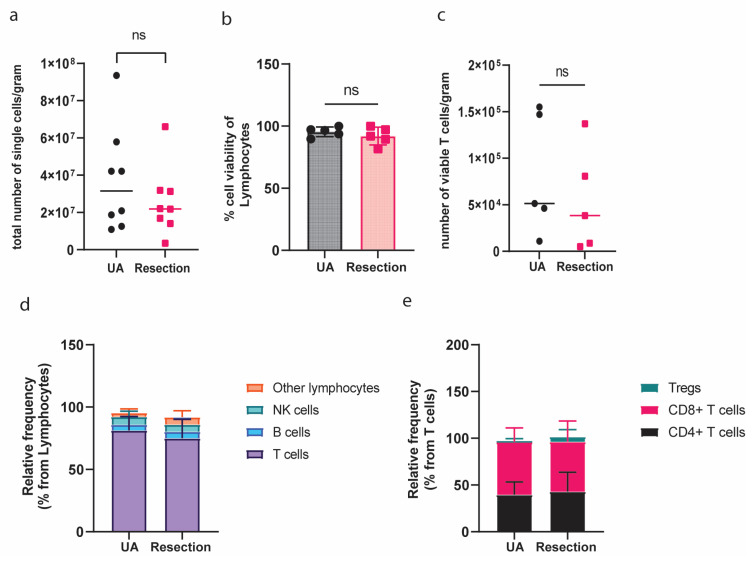
Ultrasonic aspiration-obtained cells exhibit comparable yield and viability, as well as abundance of tumor-infiltrating T, B, and NK lymphocytes compared to resection sample-derived cells. (**a**) Total number of single cells after dissociation of UA and resection (n = 8); (**b**) % Cell viability of UA- and resection-derived lymphocytes (n = 5); (**c**) Viable T-cell number retrieval per gram after dissociation of UA and resection tissue (n = 5); (**d**) Relative frequency of CD3+ T cells, CD19+ B cells, CD3-CD56+ NK cells, and other lymphocytes retrieved from UA- and resection-derived tissues (n = 5); (**e**) Relative frequency of Tregs, CD4+, and CD8+ T cells in UA- and resection-derived tissues.

To further evaluate the utility of UA, we conducted a detailed analysis of cell counts from matching tumor locations. Paired samples of UA and resected tissue were collected specifically from viable contrast-enhancing areas of eight glioblastoma tumors (Supplementary Table S1). As depicted in Figure 1A, the total numbers of single total cells per gram of tissue obtained by resected tissue and by UA did not significantly differ (*p* = 0.8). Since the viability of cells can affect their performance in downstream applications, we evaluated the cell viability of lymphocytes after dissociation of tissue derived from each of the surgical sources (n = 5) using live-dead staining (Figure 1B). This analysis showed a similar percentage of viable lymphocytes from the two tissue sources: 91.9% (range 81.6–99.7%) from resection material and 96.6% (range 89.6–99.9%) from UA-derived material (*p* > 0.05). Furthermore, we evaluated the recovery of viable T cells per gram of tissue obtained from both tissue sources. No statistically significant difference in the number of viable T cells per gram tissue was observed (*p* = 0.6) (Figure 1C).

To determine whether UA is representative of the composition of the lymphocyte compartment in resection material, we investigated the relative frequencies of T-, B-, and Natural Killer (NK) cells in single-cell suspensions derived from both tissue sources. Our findings indicated a similar distribution of these lymphocytic populations in both surgical samples (*p* > 0.05), with T cells being the predominant type (Figure 1D). Specifically, CD4, CD8 T cells, and regulatory T cells (Tregs) showed comparable abundances in UA and resection samples (Figure 1E), suggesting that UA adequately reflects the tumor tissue for these T-cell subsets.

### 3.2. Phenotypic Characteristics of T Cells Are Retained in UA-Derived Tissue

**Figure 2:**
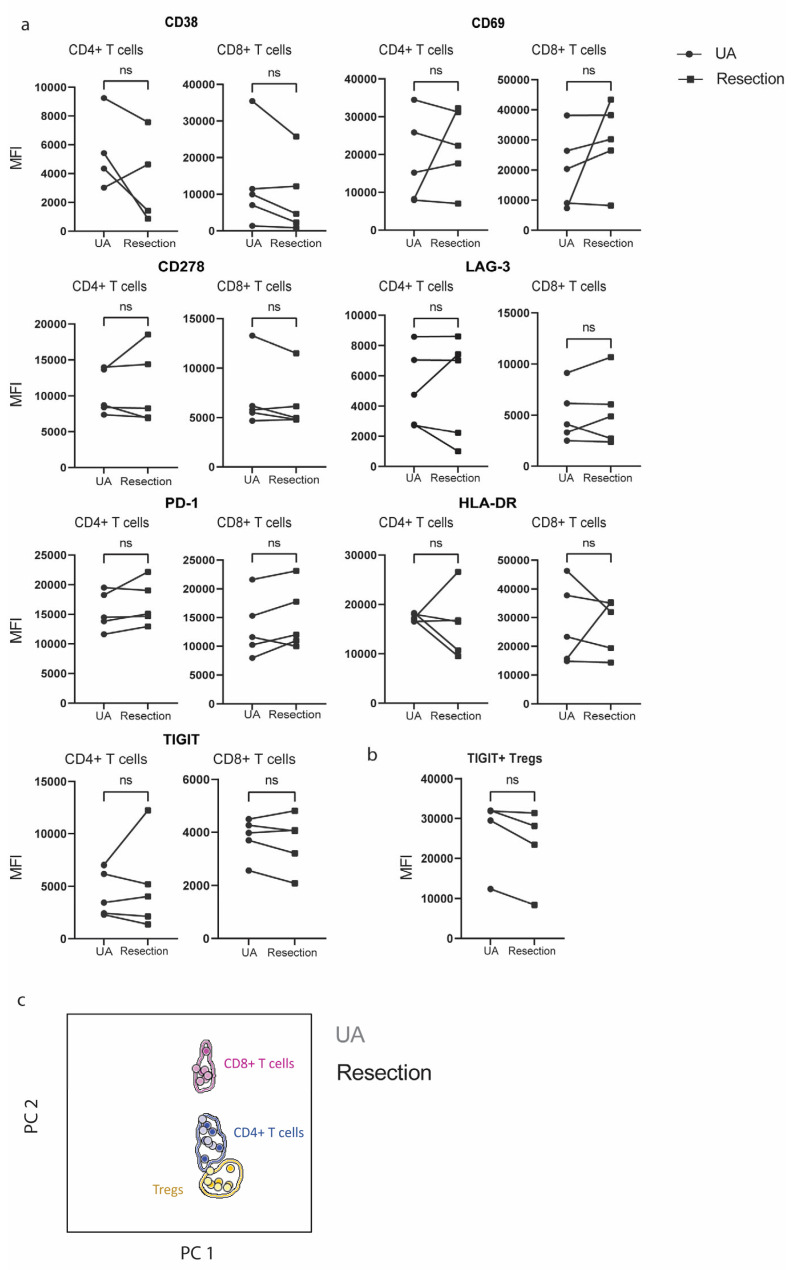
CD4+ and CD8+ T cells obtained from UA and resection exhibit similar immunophenotypic features. (**a**) Paired mean fluorescent intensity of CD38, CD69, CD278, LAG-3, PD1, TIGIT, and HLA-DR in CD4+ and CD8+ T cells retrieved from UA- and resection-derived tissues (n = 5); (**b**) Paired mean fluorescent intensity of TIGIT on CD4 + CD25 + CD127(-/low) Tregs retrieved from UA- and resection-derived tissues (n = 4); (**c**) Canonical multivariate analysis employing CD3, CD8, CD4, CD38, CD127, HLA-DR, CD278, PD1, CD69, CD45, TIGIT, and LAG-3, for overall discrimination of CD4+ T cells, CD8+ T cells, Tregs deriving from paired resection and UA tissues (n = 5). Solid circles in canonical analysis represent median values for the parameters evaluated, and solid lines depict the 1.5 standard deviation for each population identified in resection samples.

To explore whether the distinct mechanical stress that UA induces to the tissue could have impacted the T-cell phenotype, we investigated the expression of a range of co-inhibitory molecules (Programmed cell death protein (PD1), LAG-3, TIGIT) and activation markers (CD69, Human Leukocyte antigen (HLA-DR), CD278, CD38) (Figure 2a). When looking at single marker-positive CD4+ or CD8+ T cells, we found that the level of expression for all markers was similar between the paired tissue sources. For example, PD1 expression was consistently expressed at similar MFIs in both UA- and resection-derived CD4+ and CD8+ T cells. Moreover, in one of the paired samples, CD38 expression was not detected in CD4+ cells, neither in UA nor in the resected sample. Of note, the only difference we observed was in the tumor of one patient (patient GS.1225), where CD69 expression on CD4 and CD8 T cells was higher in the resected tissue compared to the UA tissue (Supplementary Figure S3). Furthermore, we also evaluated the TIGIT expression on Tregs, which was comparable between the paired tissue sources (Figure 2b). Of note, in one case no Tregs were retrieved from either tissue source. Finally, we performed canonical multivariate analysis, which revealed that the population discrimination and protein expression patterns for CD4+ T cells, CD8+ T cells, and Tregs of resection samples fully overlap with UA samples (Figure 2c).

### 3.3. Spatial Analysis of the Tumor Immune Microenvironment

**Figure 3:**
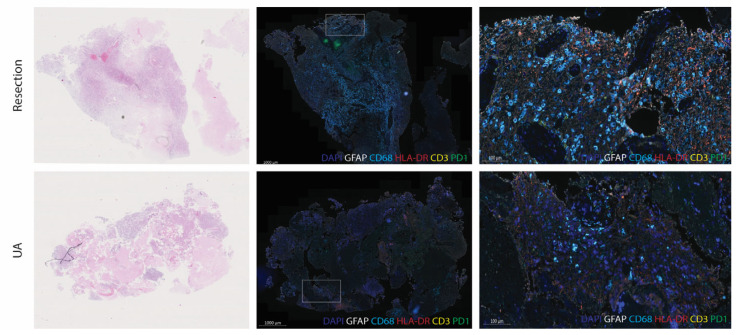
The spatial tumor immune architecture from paired resection and UA tissues. Representative hematoxylin and eosin stain (H&E) (left) and multiplex immunofluorescence (IF) images from case GS1227. Both resection- and UA-derived tissues contain cell-rich areas reflected as GFAP+ cells (Cy5/white) with a high presence of CD68+ cells (DCC/light blue) and MHC class II expression with HLA-DR (Red610/red). In the higher magnification image, CD3+ lymphocytes (R6G/yellow) expressing PD1 (FAM/green) are visible.

**Figure 4:**
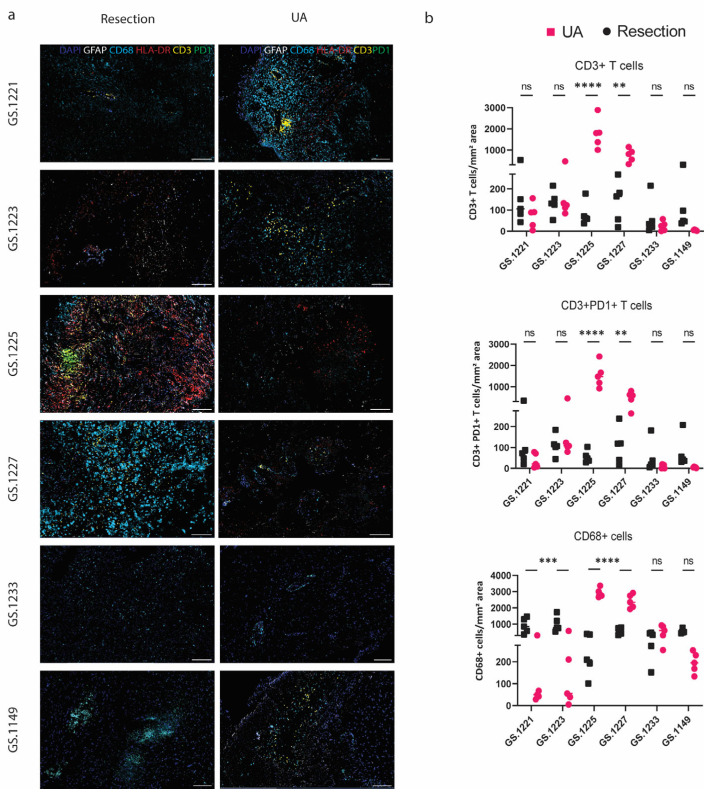
The spatial tumor immune architecture from paired resection and UA tissues. (**a**) Representative IF images of CD3+ T cells (R6G/yellow), GFAP+ cells (Cy5/white), PD1+ (FAM/green) and HLA-DR+ cells (Red610/red), and CD68+ cells (DCC/light blue) in paired resection-UA tissues from cases GS1221, GS.1223, GS1225, GS.1227, GS1233, and GS.1149. Scale bars 200 μm; (**b**) The absolute numbers of CD3+ lymphocytes and PD1+ CD3+ lymphocytes, CD68+ cells per mm^2^ tumor area (n = 6) in resection tissue compared to corresponding UA fragments for cases GS.1221, GS.1223, GS.1225, GS.1227, GS.1233, and GS.1149. Statistical significance between groups was evaluated by two-way ANOVA with correction for multiple comparisons using Sidak method (** *p* < 0.01; *** *p* < 0.005; **** *p* < 0.001).

To determine whether the spatial architecture of UA-derived and resected tissue is comparable in terms of general immune cell subsets, we conducted multiplex immunohistological analyses in paired resected and UA tissues. Our results reveal extensive intratumoral heterogeneity, both in terms of histopathology and immune cell infiltrates in both resection- and UA-derived tissues. Hematoxylin and eosin (H&E) stainings highlight regions rich in tumor cells alongside areas characterized by necrosis, a hallmark feature of high-grade gliomas (Figure 3). The multiplex IF images confirm cell-rich regions with CD68+ cells and HLA-DR+ immune cells as well as scattered T cells in both resection and UA tumors (Figure 4a). In contrast to flow cytometry-based evaluations of immune cell counts, immunofluorescence analysis detected differences in cell infiltrate densities between UA and resection samples. Specifically, cases GS.1225 and GS.1227 exhibited significantly higher T-cell and CD68+ cell counts in resected tissues, while cases GS.1221 and GS.1223 showed higher CD68+ cell numbers in UA compared to resection tissues (Figure 4b). The remaining samples displayed similar immune cell counts between the two sources. Notably, the observed differences were influenced by sample cellular density, emphasizing the impact of sample selection on IF analysis results.

## 4. Discussion

Material from intracranial surgeries is obtained by two methods: resection of solid tissue blocks and UA, the latter yielding a mixture of rinse fluid, blood, and dispersed tissue fragments. In intracranial surgeries, neurosurgeons typically employ a combination of these two techniques, a choice influenced by factors such as the tumor’s nature and location, the surgeon’s preferences, and various variables encountered during the surgical procedure. While the study of immune infiltrates of the tumor microenvironment has generally relied on resected tissue samples, material obtained from resection is less abundant in comparison to UA, thereby constraining the quantity of immune cells that can be obtained solely from resected material.

Unfortunately, however, UA material is often discarded without being utilized for further analysis, despite evidence supporting its reliability as a source for tumor cells and lymphocytes [6,7,9,13,14,15]. Immunologists often hesitate to utilize UA sources, assuming that blood contamination from the UA technique might impact the T-cell pool, rendering it difficult to distinguish between peripheral and tissue-resident T cells. It is noteworthy, however, that the UA material utilized in this study was collected through a mesh with large pores, ensuring that peripheral T cells were effectively rinsed out during the surgical procedure (Supplementary Figure S1).

Interestingly, both in our hospital and in other medical centers, UA material is routinely used alongside resected material for standard diagnostic procedures. This practice is crucial for encompassing the complete spectrum of heterogeneous areas within the tumor, facilitating accurate tumor grading [16]. Histological assessments have demonstrated that UA tissues may encompass additional and more representative features not observed in resection tissues [17] underscoring the importance of including UA material to mitigate potential sampling biases.

In light of these considerations, our study interrogated both sources, with a specific focus on characterizing the phenotype of T cells originating from the non-necrotic core of the tumor within the same patients. This emphasis is grounded in the extensively documented heterogeneity of glioblastomas, wherein significant variations in the immune phenotype are observed between the core and periphery of the tumor [18]. To validate the utility of UA-acquired tissue, it was imperative to analyze corresponding tumor locations rather than assessing the entire material obtained through each source. This approach ensures that immune differences arising from tumor heterogeneity do not impact the results concerning the utility of UA-acquired immune infiltrates as a reliable alternative to resection-acquired immune infiltrates. Our initial findings revealed that the total number of single cells and the yield of viable T cells obtained from both surgical sources are comparable. Additionally, our results demonstrate that UA-derived lymphocytes encompassing T-, B-, and NK cells are viable and exhibit a distribution that is similar to the lymphocytes derived from resection samples. These findings align with a previous study highlighting a robust correlation between resected tissues and UA tissues in terms of the CD4:CD8 ratio and the percentage of Tregs [9].

To further extend on these findings, we assessed the immune phenotype of CD4+ and CD8+ T cells in the two tissue sources and concluded that the ultrasonic aspiration process does not impact the phenotype of these cells. While it is established that enzymatic processing with collagenase IV for single-cell isolation can potentially digest certain markers on immune cells, it has not yet been investigated whether the mechanical stress produced by UA could also have a similar effect [19]. The UA technique is based on ultrasonic waves that generate energy to fragment and aspirate the tumor tissue [3], and here we show that this mechanical stress does not induce differential expression of the tested markers on CD4+ or CD8+ T cells compared to tissues obtained via traditional resection. Nevertheless, it is worth noting that our marker evaluation was limited in scope, therefore proteomic studies on T cells may provide a more comprehensive assessment of whether mechanical stress has any impact on these immune cells.

Using immunohistological imaging, we successfully demonstrated preserved cellular architecture in UA-derived tissues, specifically for tumor cells, T-cell subsets, and myeloid cells. This aligns with previous studies indicating UA’s diagnostic potential [20,21]. Interestingly, variability in immune cell densities was observed across samples, even though tissues were obtained from corresponding locations within the glioblastoma core. For instance, some resected tissue samples exhibited higher CD68+ cell counts compared to their paired UA-derived samples, while others showed the opposite trend. This variability may partly reflect the capacity of ultrasonic aspiration to sample tissues from slightly different microenvironments despite targeting similar regions. Additionally, the IF technique used for cell-density quantification examines small, localized areas of tissue, potentially amplifying the effects of intratumoral heterogeneity and leading to perceived differences in immune cell densities. Notably, flow cytometry analyses of immune cell populations did not reveal significant differences between paired samples, further suggesting that the variability seen in IF imaging might stem from methodological artifacts rather than true biological divergence. Combining UA tissues with immune cell isolation and flow cytometry could offer a more comprehensive assessment, providing a deeper understanding of immune cell dynamics throughout the entire glioblastoma microenvironment. However, it is important to acknowledge the limitations of this study. The sample size (n = 5) restricts the statistical power and generalizability of our findings. However, our paired study design and robust statistical methods help address these limitations. To confirm these results, future studies with a larger cohort are essential.

## 5. Conclusions

Taken together, our findings underscore the representativeness of UA-derived tissue as a valuable resource for conducting immunophenotypic, transcriptomic, proteomic, or functional analyses of T cells. Importantly, the process of ultrasonic aspiration does not compromise the viability or the activation status of T cells in glioblastoma. Additionally, our immunohistological analyses confirm that UA-derived tissue maintains its tumor and immune cell architecture, enabling the evaluation of diverse regions within the tumor. The relative abundance of CUSA-derived tissue further enhances its utility, providing ample material for comprehensive analyses and experimental reproducibility. Thus, UA-derived tissue proves to be a suitable alternative to resection tissue for conducting T-cell phenotype experiments and investigating the interactions between T cells and other cells within the GBM microenvironment.

## Data Availability

The original contributions presented in the study are included in the article/Supplementary Materials, further inquiries can be directed to the corresponding author.

## Supplementary Materials

The following supporting information can be downloaded at: https://www.mdpi.com/article/10.3390/cancers17040603/s1, Supplementary Figure S1: Collection of Ultrasonic Aspiration (UA) tissue fragments (a) The mesh in the CUSA Excel+ features large pores (b) The mesh is positioned in the CUSA Excel+, collecting UA tissue pieces while blood is rinsed out (c) The UA material is retained in the mesh during transport to the laboratory (d) The mesh is carefully opened with scalpel knives to extract UA tissue fragments. Supplementary Figure S2: Ultrasonic aspiration tissue is more abundant than resection tissue. The total weight in grams of glioblastoma tissue acquired with either UA (n = 15) or resection (n = 17). Supplementary Figure S3: CD69 expression on CD4+ and CD8+ T cells in case GS.1225. Fluorescence histograms showing CD69 expression on CD4+ T cells (pink) and CD8+ T cells (yellow) retrieved from UA- and resection-derived tissues. Supplementary Table S1: Clinical characteristics of the GBM patients. Supplementary Table S2: Flow cytometry antibodies. Supplementary Table S3: IF antibodies.
