# Supplementary material for: Ultrasonic Aspiration-Acquired Glioblastoma Tissue Preserves Lymphocyte Phenotype and Viability, Supporting Its Use for Immunological Studies
Source: Cancers (Basel). 2025 Feb 11;17(4):603. doi: 10.3390/cancers17040603 (PMC11853073; doi:10.3390/cancers17040603)
Supplement: Supplementary file 1 [file cancers-17-00603-s001.zip › cancers-3304405-supplementary-tables.pdf]

Table S1: Clinical characteristics of the GBM patients

GS ID	Histology	IDH	MGMT	Driver mutations
**1233**	GBM	WT	unknown	TERT promoter; CDKN2A/B, PTEN
**1227**	GBM	WT	methylated	N/A
**1225**	GBM	WT	methylated	N/A
**1223**	GBM	WT	methylated	EGFR exon 7 amplification
**1221**	GBM	WT	methylated	TERT promoter; CDKN2A; TP53; loss of 10q and 13q
**1149**	GBM	WT	unknown	N/A
**1077**	GBM	MT	methylated	SUFU amplification

Table S2: Flow cytometry antibodies

Target (Hu)	Fluorochrome	Clone	Company	Cat no.
**CD14**	BUV395	M5E2	BD Biosiences	740286
**CD56**	BUV563	NCAM16.2	BD Biosiences	612928
**CD3**	BUV661	UCHT1	BD Biosiences	612964
**CD19**	BUV737	HIB19	BD Biosiences	741829
**CD8**	BUV805	RPA-T8	BD Biosiences	749366
**CD38**	PacB	HB-7	BioLegend	256628
**HLA-DR**	BV570	L243	BioLegend	30763
**CD69**	BV785	FN50	BD Biosiences	563834
**PD1**	BV711	EH12.1	BD Biosiences	564017
**CD278**	BV650	DX29	BD Biosiences	563832

**LAG-3**	PE-Cy7	11C3C65	Biolegend	369309
**CD45**	AF532	HI30	Thermo Fisher Scientific	58-0459-42
**TIGIT**	PE-Dazzle594	A15153G	BioLegend	372715
**CD4**	SparkNIR685	SK3	BD Biosiences	344657
**LIVE/DEAD**	Blue		Thermo Fisher Scientific	L23105

Table S3: IF antibodies

**Target**	**Type**	**Clone**	**Company**	**Cat. Number**
**CD3**	Anti-rabbit	2GV6	Ventana	790-4341
**CD68**	Anti-mouse	KP-1	Ventana	790-2931
**HLA-DR**	Anti-mouse	CR3/43	DAKO	M0775
**GFAP**	Anti-rabbit	28E1	Abcam	Ab20334
**PD-1**	Anti-mouse	NAT105	Cell Marque	760-4895
