# Supplementary figures and images for: Ultrasonic Aspiration-Acquired Glioblastoma Tissue Preserves Lymphocyte Phenotype and Viability, Supporting Its Use for Immunological Studies
Source: Cancers (Basel). 2025 Feb 11;17(4):603. doi: 10.3390/cancers17040603 (PMC11853073; doi:10.3390/cancers17040603)

Supplementary Figure S1

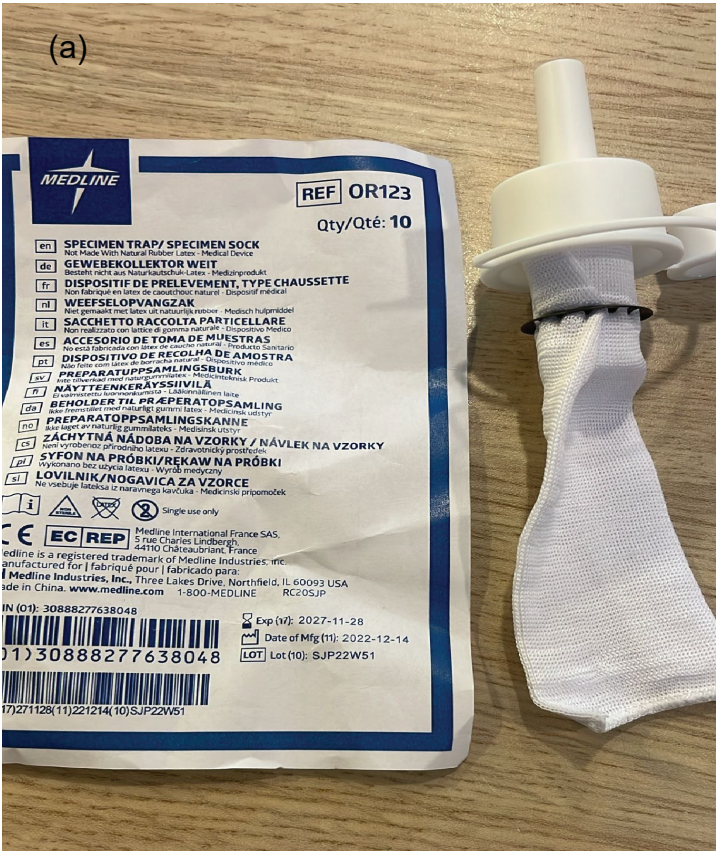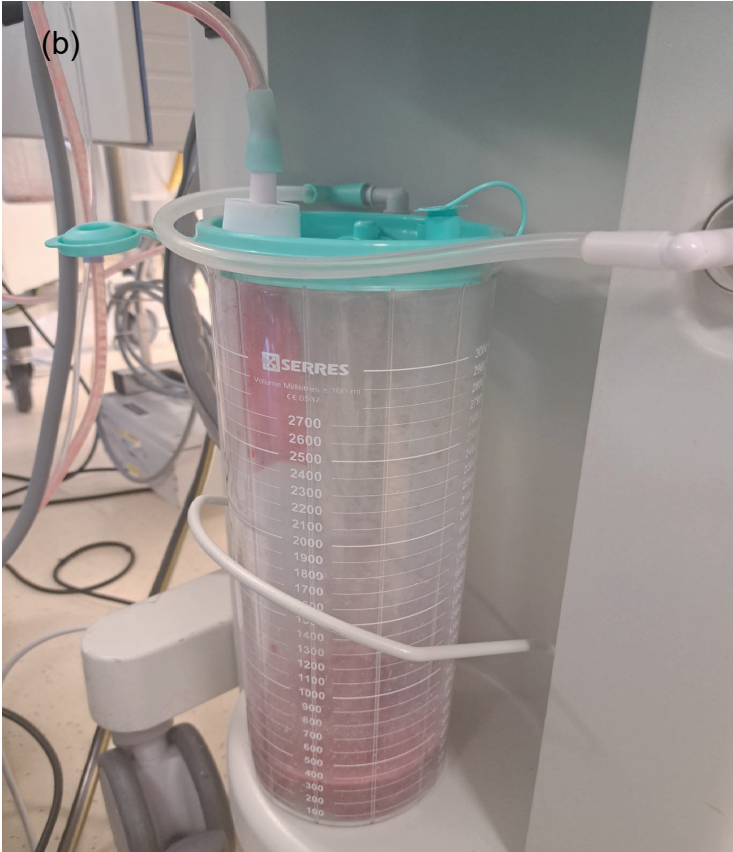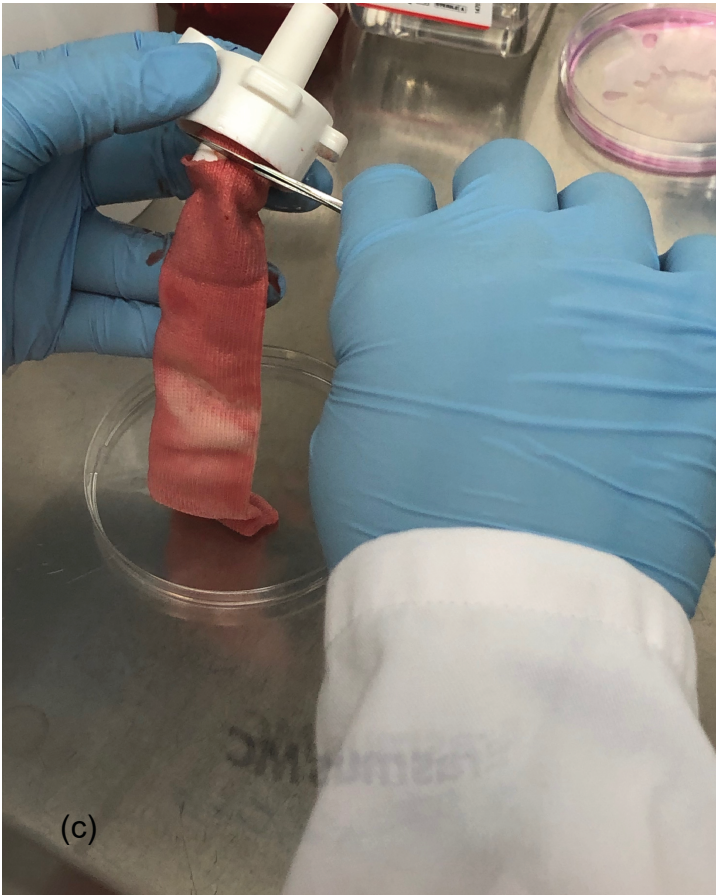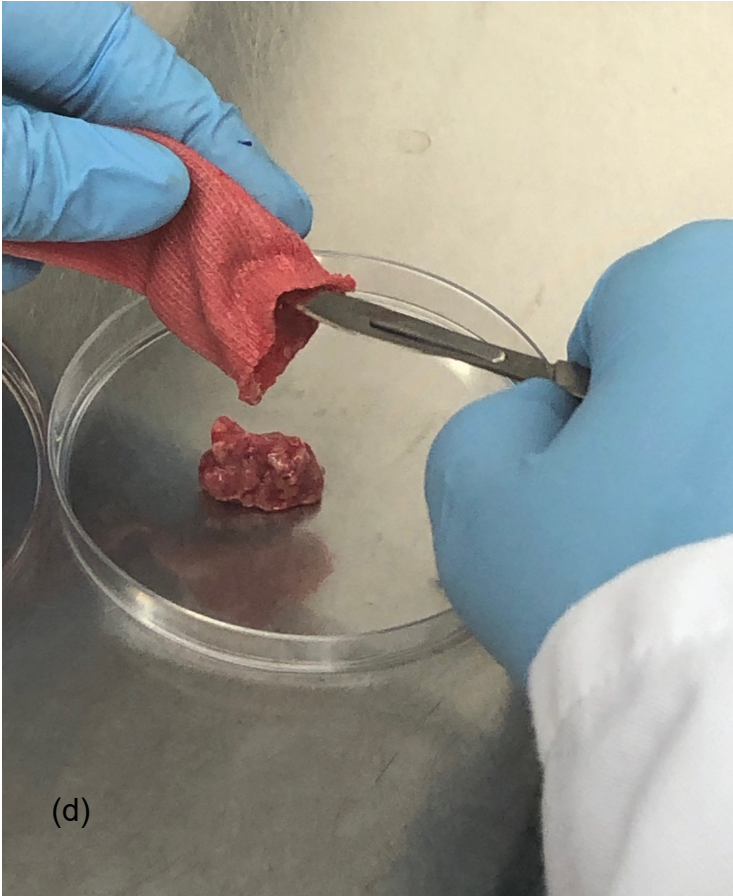

Supplementary Figure S2

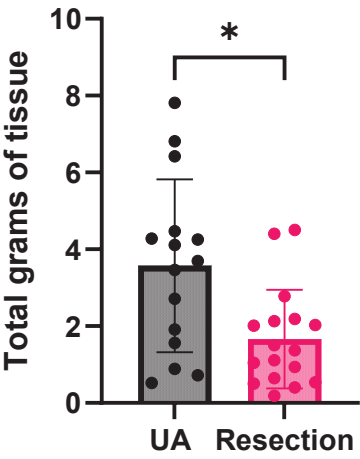

Supplementary Figure S3

GS.1225

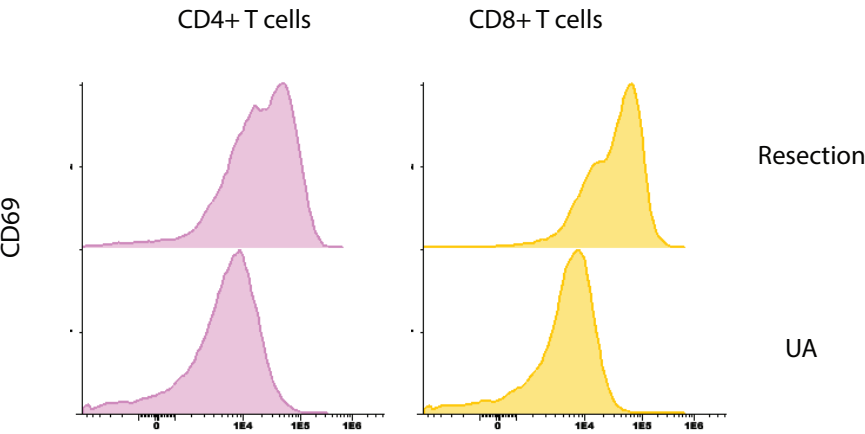

Supplement: Supplementary file 1 [file cancers-17-00603-s001.zip › cancers-3304405-supplementary-figures.pdf]
